# Oxaliplatin regulates myeloid‐derived suppressor cell‐mediated immunosuppression via downregulation of nuclear factor‐κB signaling

**DOI:** 10.1002/cam4.1878

**Published:** 2018-12-27

**Authors:** Na‐Rae Kim, Yeon‐Jeong Kim

**Affiliations:** ^1^ Laboratory of Microbiology and Immunology, College of Pharmacy Inje University Gimhae Korea; ^2^ Inje Institute of Pharmaceutical Science and Research Inje University Gimhae Korea

**Keywords:** anticancer agent, immunosuppression, myeloid‐derived suppressor cell, NF‐κB, oxaliplatin

## Abstract

Myeloid‐derived suppressor cells (MDSCs) represent one of the major types of immunoregulatory cells present under abnormal conditions, including cancer. These cells are characterized by their immature phenotype and suppressive effect on various immune effectors. In both human and mouse, there are two main subsets of MDSCs: polymorphonuclear (PMN)‐MDSCs and mononuclear (Mo)‐MDSCs. Thus, strategies to regulate MDSC‐mediated immunosuppression could result in the enhancement of anticancer immune responses. Oxaliplatin, a platinum‐based anticancer agent, is widely used in clinical settings. It is known to induce cell death by interfering with double‐stranded DNA and interrupting its replication and transcription. In this study, we found that oxaliplatin has the potential to regulate MDSC‐mediated immunosuppression in cancer. First, oxaliplatin selectively depleted MDSCs, especially Mo‐MDSCs, but only minimally affected T cells. In addition, sublethal doses of oxaliplatin eliminated the immunosuppressive capacity of MDSCs and induced the differentiation of MDSCs into mature cells. Oxaliplatin treatment diminished the expression of the immunosuppressive functional mediators arginase 1 (ARG1) and NADPH oxidase 2 (NOX2) in MDSCs, while an MDSC‐depleting agent, gemcitabine, did not downregulate these factors significantly. Oxaliplatin‐conditioned MDSCs had no tumor‐promoting activity in vivo. In addition, oxaliplatin modulated the intracellular NF‐κB signaling in MDSCs. Thus, oxaliplatin has the potential to be used as an immunoregulatory agent as well as a cytotoxic drug in cancer treatment.

## 1 INTRODUCTION

Chemotherapeutic agents that target cancer cells directly are the foundation of cancer therapy. There are several types of chemotherapeutic agents, including DNA‐alkylating agents, intercalating agents, and antimicrotubule agents. Although these drugs are effective against highly proliferative cancer cells, they also lead to cytotoxic effects on proliferating immune effectors and normal tissues.^1^

Recently, it has been suggested that chemotherapeutic drugs do not only kill proliferating tumor cells but also contribute to the stimulation of antitumor immune responses as immunogenic antigens are released from dead tumor cells.^1^ Furthermore, some anticancer drugs have been found to act as immunomodulators.^2^ In particular, the treatment of myeloid lineage cells, such as dendritic cells (DCs) and monocytes, with various cytotoxic drugs results in immune activation. Treatment with docetaxel,^3^ topotecan,^4^ or vinca alkaloids^5^ increases DC maturation and augments DC immunogenicity.

Oxaliplatin is a platinum‐based anticancer drug that blocks DNA replication and transcription by binding to double‐stranded DNA, especially guanine bases, resulting in cytotoxic effects in proliferating cells.^6^ This agent differs from other platinum compounds, such as cisplatin and carboplatin, in terms of its intracellular targets and mechanism of resistance.^6^, ^7^ Oxaliplatin‐based therapy is effective and well tolerated and has been used as a first‐line therapy against advanced colorectal cancer.^8^

In addition to its direct cytotoxic effects on cancer cells, oxaliplatin treatment modulates the immunosuppressive tumor environment. Oxaliplatin downregulates the expression of the inhibitory protein programmed death receptor‐ligand 2 (PD‐L2) on DC and tumor cells, resulting in more efficient tumor‐specific T‐cell responses.^9^ In contrast, oxaliplatin increases the expression of PD‐L1 on plasmacytoid DCs and weakens their immunogenicity.^10^ Despite the interesting immunoregulatory capacity of oxaliplatin, previous studies have limited their focus to immune effector cells, particularly DCs.^9^, ^10^ Herein, we focused on another myeloid lineage cell, myeloid‐derived suppressor cells (MDSCs).

Myeloid‐derived suppressor cells were originally defined as myeloid lineage cells that acquired immunosuppressive functions under pathological conditions.^11^ They inhibit antitumor immune effectors via several mechanisms. In particular, Arg‐1, NOX2, and iNOS are recognized as functional mediators of MDSCs in immunosuppression. Based on preclinical and clinical data that show the accumulation of MDSCs in blood, bone marrow, and tumor sites and their suppressive activity against antitumor immune responses, various strategies for targeting MDSCs have been proposed for cancer treatment.^11^, ^12^

Anticancer cytotoxic drugs have been used to deplete MDSCs and induce reductions in MDSC levels. In the present study, we confirmed the selective depletion of MDSCs following oxaliplatin treatment in vivo and in vitro, and by extension, the effect of a less cytotoxic dose of oxaliplatin on MDSCs. Oxaliplatin reduced the suppressive function of MDSCs by inhibiting nuclear factor κ B (NF‐κB) signaling. These results may be instrumental in identifying new therapeutic mechanisms in oxaliplatin‐treated cancer patients.

## 2 MATERIALS AND METHODS

### 2.1 Mice

Specific pathogen‐free BALB/c mice were purchased at 5 weeks of age from Orient bio ( Sungnam, Korea), and specific pathogen‐free DO11.10 mice were kindly gifted by Dr Kang Chang‐Yuil in Seoul National University. All mice were housed at the Animal Resource Center of Inje University. Experiments were approved by the Institutional Animal Care and Use Committee of Inje University (Approval number: 2015‐9).

### 2.2 Tumor model

To establish a mouse tumor model, 2 × 10^5^ CT26 tumor cells were subcutaneously (sc) injected into the left flank of each BALB/c mouse. For isolation of MDSCs, when the tumor size reached approximately 1500 mm^3^, tumor‐bearing mice were sacrificed. In a previous study, we confirmed that approximately 40 days were required to establish a solid tumor mass of 1500 mm^3^ and that at that point, more than 20% of the splenocytes were MDSCs that expressed surface CD11b and Gr‐1 and had suppressive abilities.^14^ Tumor size was measured by caliper three times per week and was calculated as follows: the longest length × the shortest width × height × *π*/6. Tumor‐bearing mice were monitored and sacrificed before severe lung metastasis or solid tumor necrosis for humanitarian reasons.

### 2.3 Cell lines

CT26 cells (Korean Cell Line Bank, Seoul, Korea) were maintained in Dulbecco's modified Eagle's medium (DMEM) supplemented with 10% fetal bovine serum (FBS) and 1% penicillin‐streptomycin solution (all from Gibco BRL, Invitrogen Life Technologies, Darmstadt, Germany).

### 2.4 Antibodies (Abs) and flow cytometry

To detect T‐cell populations, fluorescein isothiocyanate (FITC)‐labeled anti‐CD3 Abs, phycoerythrin (PE)‐labeled anti‐CD8 Abs, and PE‐labeled anti‐CD4 Abs were purchased from BioLegend (San Diego, CA, USA). We used a Forkhead Box P3 (Foxp3) Staining Buffer Set and allophycocyanin (APC)‐conjugated anti‐FoxP3 Abs (both from eBioscience, Waltham, MA, USA) and performed intranuclear staining according to the manufacturer's instructions. To identify the two main subsets of MDSCs, anti‐CD11b Abs conjugated with FITC, anti‐Ly‐6G Abs conjugated with APC, and anti‐Ly‐6C Abs conjugated with PE (BioLegend) were used. For analysis of MDSC phenotypes, isolated CD11b^+^ cells were stained with anti‐Ly‐6C Abs conjugated with PE, anti‐Ly‐6G Abs conjugated with FITC, as well as APC‐labeled anti‐CD40, anti‐IA/IE, anti‐PD‐L1, anti‐F4/80, or anti‐CD11c Abs (all from BioLegend). Cells stained with fluorescent Abs were detected using a FACSCaliber flow cytometer (BD Biosciences, San Jose, CA, USA). For isotype control staining, we used APC‐conjugated rat IgG2a, rat IgG2b, and hamster IgG (all from BioLegend).

For the staining of phosphorylated NF‐κB, CD11b^+^ cells were isolated from the spleens of tumor‐bearing mice using the MACS system and incubated in lipopolysaccharide (LPS) containing serum‐free RPMI 1640 medium in the presence or absence of oxaliplatin for 4 h. After incubation, cells were stained with PE‐conjugated Ly‐6C Abs, FITC‐conjugated Ly‐6G Abs (both from BioLegend), and Alexa Fluor 647‐conjugated phospho‐NF‐κB Abs (Cell Signaling Technology, Danvers, MA, USA) using the Transcription Factor Phospho Buffer Set (BD Biosciences), according to the manufacturer's recommendations. Phosphorylated NF‐κB expression was assessed by flow cytometry.

### 2.5 Cell isolation

To purify MDSCs, splenocytes from tumor‐bearing mice were stained with anti‐CD11b microbeads (Miltenyi Biotec, Bergisch Gladbach, Germany) and enriched by positive selection using the MACS technique (Miltenyi Biotec). To obtain DO11.10 T cells, CD4^+^ T cells were isolated via negative selection using a CD4^+^ T‐cell isolation kit (Miltenyi Biotec).

For the isolation of tumor infiltrating leukocytes (TILs), solid tumors were isolated from CT26 tumor‐bearing mice. The tumors were fragmented and digested with collagenase D (Roche, Basel, Swiss) and DNase I (Roche) using the GentleMACS dissociator (Miltenyi Biotec), according to the manufacturer's recommendation. Subsequently, the cells were separated using 40%/70% Percoll (GE Healthcare Life Sciences, Chicago, IL, USA) gradient and leukocytes were obtained from the interphase.

### 2.6 In vivo depletion study

BALB/c mice were sc injected with 2 × 10^5^ CT26 cells. Thirty‐four days after tumor challenge, when the tumor size reached approximately 1200 mm^3^, drug treatment was started. The first group was intraperitoneally (ip) treated with 10 mg/kg of oxaliplatin (Sigma, Darmstadt, Germany), a dose that was mildly effective at inhibiting tumor growth in a mouse xenograft model.^15^ The second group was ip injected with 120 mg/kg of gemcitabine (Sigma), dose that was effective at depleting the MDSC population in a previous study.^16^ Phosphate‐buffered saline (PBS, Gibco BRL, Invitrogen Life Technologies) was used as a control. After 48 hours, mice were sacrificed to assess T‐cell subsets and MDSCs. Total splenocytes isolated from tumor‐bearing mice were counted for calculating absolute cell numbers and were stained with fluorescent Abs for the detection of CD8^+^ T cells, CD4^+^ T cells, regulatory T cells, or MDSC subsets.

### 2.7 In vitro viability test

Splenic MDSCs were plated at 4 × 10^5^ cells/well, and serial dilutions of oxaliplatin or gemcitabine were added to cells in the presence of LPS (Sigma). After 24 hours of incubation, EZ‐cytox reagent (Daeillab, Seoul, Korea), a water‐soluble tetrazolium (WST) salt, was added to the wells. After another 4 hours of incubation, absorbances were read at 450 nm using an ELISA reader (Sunrise, Tecan, Männedorf, Switzerland). We calculated the percentage of cell viability as: [(optical density (OD) of well containing treated MDSCs − blank OD)/(OD of well containing untreated MDSCs − blank OD)] × 100. The blank OD was defined as the absorbance of a well containing culture medium only. The means of triplicate experiments were determined.

### 2.8 Quantitative real‐time PCR

Splenic MDSCs were plated at 5 × 10^6^ cells/well in a 12‐well plate and incubated with the indicated concentration of oxaliplatin or gemcitabine in the presence of LPS (100 ng/mL) for 24 hours. Sterile ultrapure water (Biosesang, Sungnam, Korea) was used as a vehicle. Total RNA was isolated from MDSCs using the RNeasy Mini Kit (Qiagen, Dusseldorf, Germany) and was used as a template in a reverse transcription reaction to obtain complementary DNA (cDNA) using M‐MLV reverse transcriptase (Enzynomics, Daejeon, Korea). Quantitative real‐time PCR was performed using TOPreal™ qPCR 2 × PreMIX (SYBR Green) (Enzynomics). Expression levels of the genes of interest were normalized to GAPDH levels for each sample. The value of the relative expression of the vehicle treated sample was set to 1, to which the values of relative expression of other samples were normalized. The following primers (all from Cosmogenetech, Daejeon, Korea) were used: *ARG1*, forward 5ʹ‐AAC ACG GCA GTG GCT TTA ACC T‐3ʹ, reverse 5ʹ‐ GTG ATG CCC CAG ATG GTT TTC‐3ʹ; *iNOS*, forward 5ʹ‐AGG AAG TGG GCC GAA GGA T‐3ʹ, reverse, 5ʹ‐GAA ACT ATG GAG CAC AGC CAC AT‐3ʹ; *NOX2*, forward 5ʹ‐GAC CCA GAT GCA GGA AAG GAA‐3ʹ, reverse 5ʹ‐TCA TGG TGC ACA GCA AAG TGA T‐3ʹ; *GAPDH*, forward 5ʹ‐CCT GGA GAA ACC TGC CAA GTA T‐3ʹ, reverse 5ʹ‐GGA AGA GTG GGA GTT GCT GTT G‐3ʹ.

### 2.9 Phenotypic analysis of MDSCs

Splenic MDSCs were treated in vitro with 1 μg/mL of oxaliplatin and LPS (100 ng/mL). After 24 hours, cells were harvested and stained with fluorescent Abs.

### 2.10 In vitro T‐cell suppression

MDSCs were isolated and labeled with 10 μmol/L of chloromethylfluorescein diacetate succinimidyl ester (CFSE; Invitrogen). DO11.10 CD4^+^ T cells were purified from naïve DO11.10 mice and plated at 2 × 10^5^ cells/well in a 96‐well plate (SPL, Pocheon, Korea). CFSE‐labeled MDSCs were cocultured with DO11.10 CD4^+^ T cells with 10 μg/mL OVA peptide (Sigma) for 72 hours at a 1:1 ratio. After incubation, cells were harvested and stained with anti‐CD4 Abs conjugated with PE (BioLegend). Flow cytometric analysis was performed to detect CFSE dilution in the PE^+^ cell population.

### 2.11 MDSC adoptive transfer

Myeloid‐derived suppressor cells were treated with 1 μg/mL of oxaliplatin in the presence of 100 ng/mL of LPS for 2 hours. To identify the role of MDSCs in tumor‐bearing mice, they were injected iv (1 × 10^7^ cells/mouse) into CT26 tumor‐bearing mice. Tumor size was monitored three times a week. To analyze the immunogenicity of oxaliplatin‐treated MDSCs, they were pulsed with 10 µg/mL of Her‐2/neu CTL epitope peptide (hP63)^17^ (Anygen, Daejeon, Korea) during 2 hours of oxaliplatin treatment. CTL peptide‐pulsed MDSCs were iv injected into naïve mice (2 × 10^6^ cells/mouse). Thirteen days later, in vivo CTL responses were analyzed as described below.

### 2.12 In vivo cytotoxic T lymphocytes (CTL) assay

Splenocytes were obtained from naïve BALB/c mouse lymphocytes and pulsed with 5 µg/mL hP63 or left unpulsed for 90 minutes, and were then labeled with 18 μmol/L or 2 μmol/L CFSE, respectively. CFSE^high^ and CFSE^low^ cells were mixed equally and injected iv into the immunized mice. Twenty four hours later, CFSE^+^ cells in splenocytes were analyzed by flow cytometry. The specific lysis was calculated as follows: *r* (ratio) = (% CFSE^low^/% CFSE^high^), % specific lysis = [1 − (*r*_control_/*r*_treat_)] × 100.

### 2.13 Measurement of NF‐κB p65

Purified MDSCs were stimulated by incubation with 100 ng/mL LPS in the presence or absence of oxaliplatin for 30 minutes. After incubation, cells were collected and lysed with Cell Extraction Buffer PTR (Abcam, Cambridge, MA, USA). NF‐κB p65 total protein and phosphorylated NF‐κB p65 (pS536) were measured by SimpleStep ELISA kit (Abcam), according to the manufacturer's recommendations.

### 2.14 Statistical analysis

One‐tailed Student's *t* tests were performed to compare differences between two groups using SigmaPlot 12.5 software. Values of *P < *0.05 were considered significant.

## 3 RESULTS

### 3.1 Oxaliplatin selectively depletes MDSCs but not T cells in tumor‐bearing mice

**Figure 1:**
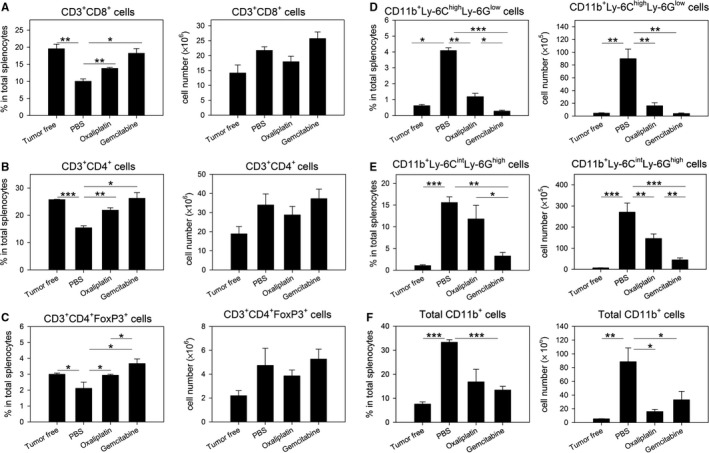
In vivo treatment with oxaliplatin selectively removed myeloid‐derived suppressor cells (MDSCs) but not T cells in tumor‐bearing mice. BALB/c mice (n = 3/group) were sc inoculated with 1 × 10^5^ CT26 cells/mouse. When the average tumor size reached approximately 1200 mm^3^, 10 mg/kg oxaliplatin or 120 mg/kg gemcitabine was ip injected into tumor‐bearing mice. Two days after drug treatment, mice were sacrificed, and T cells and MDSC subsets among total splenocytes were detected by flow cytometry. A, Percentages (left) and absolute numbers (right) of CD3^+^ CD8^+^ cells in splenocytes; B, CD3^+^ CD4^+^ cells in splenocytes; C, CD3^+^ CD4^+^ FoxP3^+^ cells in splenocytes; D, CD11b^+^Ly‐6C^high^Ly‐6G^low^ cells in splenocytes; E, CD11b^+^Ly‐6C^int^Ly‐6G^high^ cells in splenocytes; F, Total CD11b^+^ cells in splenocytes. Representative data from two separate experiments are shown. *P < 0.05, **P < 0.01, ***P < 0.001

First, we assessed the cytotoxic activity of oxaliplatin against immune effectors and immunosuppressors in tumor‐bearing mice. As a control agent, gemcitabine, which is known to be a cytotoxic drug that selectively targets MDSCs,^1^, ^16^ was used at a general dosage (120 mg/kg) to deplete MDSCs. Oxaliplatin was given ip at 10 mg/kg, a dose that has been shown to be tolerated and to exhibit cytotoxic activity against tumors.^15^ During tumor progression, percentages of CD8^+^ T cells, CD4^+^ T cells, and FoxP3^+^ Tregs were reduced in the spleen, while their absolute numbers were increased, though this difference was not significant (Figure 1A‐C). The percentages and numbers of CD11b^+^Ly‐6C^high^Ly‐6G^low^ Mo‐MDSCs and CD11b^+^Ly‐6C^int^Ly‐6G^high^ PMN‐MDSCs were markedly increased in the spleens of tumor‐bearing mice, and oxaliplatin reduced total myeloid cells and both subsets of MDSCs, especially Mo‐MDSCs (Figure 1D‐F). Gemcitabine was more cytotoxic to MDSCs than oxaliplatin at the implemented dosages. Unlike oxaliplatin treatment, PMN‐MDSCs were depleted as effectively as Mo‐MDSCs following gemcitabine treatment. Both oxaliplatin and gemcitabine treatment induced increases in the percentages of T‐cell populations, which may be a result of the reductions in MDSCs. Additionally, when treatment of oxaliplatin was performed twice at a 2‐day interval, MDSC‐depleting activity was significantly increased (Figure S1). In fact, repeated treatment with oxaliplatin (two doses) reduced both Mo‐MDSCs and PMN‐MDSCs more dramatically, with a higher depletion efficiency than that with a single treatment of gemcitabine. Collectively, oxaliplatin depleted Mo‐MDSCs and PMN‐MDSCs in tumor‐bearing mice, analogously to gemcitabine, but it did not significantly affect levels of effector CD4^+^/CD8^+^ T cells or Tregs.

### 3.2 Treatment with oxaliplatin resulted in increase of CD8^+^ T cells and CD4^+^ T cells, but decrease of regulatory T cells at the tumor site

**Figure 2:**
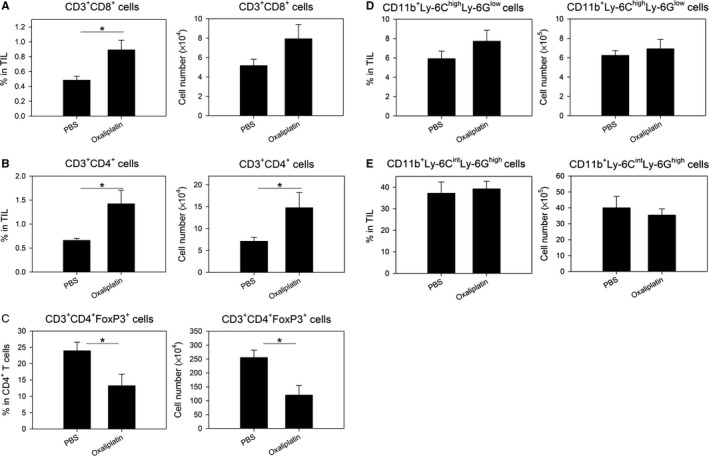
Treatment with oxaliplatin resulted in increase of immune effectors and decrease of myeloid‐derived suppressor cells at the tumor site. When the average tumor size reached about 100 mm^3^, 10 mg/kg oxaliplatin was ip injected into tumor‐bearing mice (n = 3/group). Two days after drug treatment, TILs were isolated from tumor‐bearing mice, as mentioned in Materials and Methods. TILs were stained with fluorescent‐labeled Abs and analyzed by flow cytometry. Percentages (left) and absolute numbers (right) of A, CD3^+^ CD8^+^ cells in TILs; B, CD3^+^ CD4^+^ cells in TILs; C, CD3^+^ CD4^+^ FoxP3^+^ cells in TILs; D, CD11b^+^Ly‐6C^high^Ly‐6G^low^ cells in TILs; E, CD11b^+^Ly‐6C^int^Ly‐6G^high^ cells in TILs. *P < 0.05, **P < 0.01, ***P < 0.001

Since MDSCs show their main immunosuppressive effect at the tumor site, we analyzed the changes of immune cells in TILs. As shown in Figure 1, in the spleen, the percentages of effector T cells, such as CD8^+^ T cells and CD4^+^ T cells, were increased, while the percentages of two subsets of MDSCs were decreased by oxaliplatin treatment in tumor‐bearing mice. At the tumor site, not only percentages, but also absolute cell numbers of effector T cells were significantly increased by oxaliplatin treatment (Figure 2). Regulatory T cells were reduced, but Mo‐MDSCs and PMN‐MDSCs were not changed within the 2 days of oxaliplatin treatment. Generally, distribution of drugs to the tumor site is limited.^18^ Therefore, cells at tumor site might be affected by oxaliplatin treatment indirectly, and tumor MDSCs could not be removed by oxaliplatin treatment within 2 days.

### 3.3 In vitro cytotoxicity of oxaliplatin compared with that of gemcitabine

**Figure 3:**
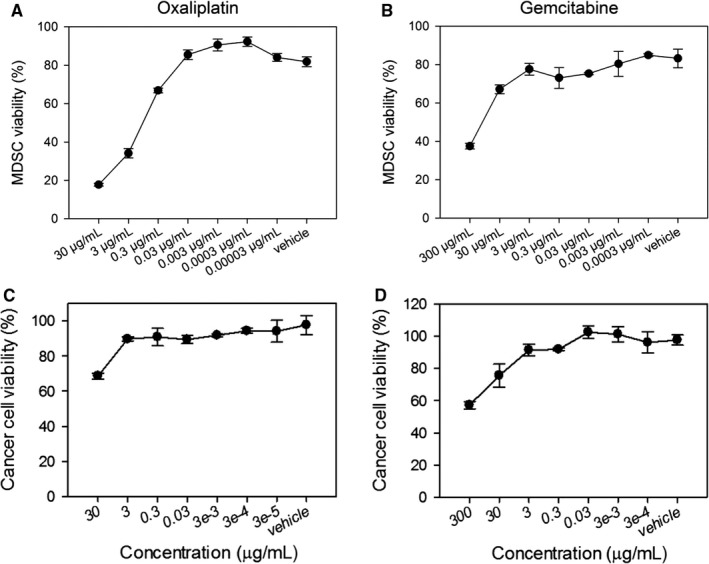
Effect of oxaliplatin on in vitro myeloid‐derived suppressor cell (MDSC) viability compared with that of the selective MDSC depletion agent gemcitabine. BALB/c mice were sc inoculated with 1 × 10^5^ CT26 cells/mouse. When the average tumor size reached approximately 1500 mm^3^, CD11b^+^ cells were isolated from splenocytes of tumor‐bearing mice. (A and B) CD11b^+^ MDSCs were seeded, and six 10‐fold serial dilutions of oxaliplatin or gemcitabine were added at concentrations starting at 30 μg/mL and 300 μg/mL, respectively. Sterile distilled water was used as a vehicle. To improve MDSC viability, 100 ng/mL LPS was also added. Viability of MDSCs treated with various concentrations of oxaliplatin (A) and gemcitabine (B) is shown. (C and D) CT26 cancer cells were seeded and incubated for 24 h. Indicated concentrations of drugs were added. After 24 h of incubation, cell viability was analyzed by formazan formation assay, and absorbances were measured at 450 nm. Viability of CT26 cancer cells treated with various concentrations of oxaliplatin (C) and gemcitabine (D) is shown. Each sample was assayed in triplicate. Representative data from two separate experiments are shown

We performed viability tests with the two cytotoxic drugs to select doses that were less cytotoxic to MDSCs. Due to the poor viability of MDSCs in vitro without supplements, we added LPS to the MDSCs. About 34% of MDSCs were viable at 3 μg/mL of oxaliplatin, and 66% of MDSCs survived at 0.3 μg/mL following a 24‐hour incubation (Figure 3A). Less than 0.03 μg/mL oxaliplatin did not significantly induce cell death in MDSCs compared with vehicle. In the case of gemcitabine, 37% of MDSCs were viable at 300 μg/mL, and 67% of MDSCs remained at 30 μg/mL (Figure 3B). Thus, oxaliplatin was much more potent than gemcitabine at the same concentration in terms of in vitro MDSC depletion. We determined that 1 μg/mL oxaliplatin or 100 μg/mL gemcitabine represented concentrations less toxic to MDSCs.

On the other hand, we analyzed the cytotoxicity of oxaliplatin and gemcitabine against CT26 cancer cells (Figure 3C,D). Treatment with each drug resulted in reduced viability of cancer cells, and the in vitro cytotoxicity against CT26 cells was lower than that against MDSCs. We found that about 20% of MDSCs survived after 30 μg/mL of oxaliplatin treatment, while 68% of cancer cells were viable under the same condition.

### 3.4 Oxaliplatin regulates immunosuppressive mediators of MDSCs

Next, we analyzed the effect of the cytotoxic agents on the expression of functional mediators of MDSCs in the presence of 100 ng/mL LPS. Among the several methods of MDSC maintenance or activation,^14^, ^19^, ^20^ we used LPS stimulation because we confirmed that LPS sufficiently activated MDSCs; a dramatically higher expression of the NOX2 gene was seen in LPS‐treated MDSCs, compared with in tumor cell conditioned medium (TCCM)‐treated MDSCs (Figure S2).

**Figure 4:**
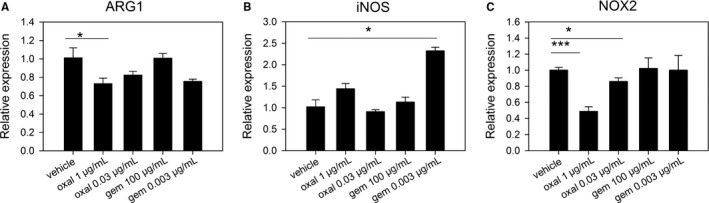
Oxaliplatin induced the downregulation of immunosuppressive mediators in MDSCs. CD11b^+^ cells were purified from the splenocytes of CT26 tumor‐bearing mice and treated with the indicated concentrations of oxaliplatin or gemcitabine in the presence of 100 ng/mL LPS. Sterile distilled water was used as a vehicle. After 24 h of treatment, total RNA was extracted from MDSCs and used as a template for cDNA synthesis. Quantitative PCR was performed to analyze the mRNA levels of *ARG1*,* iNOS*, and *NOX2*. Each sample was prepared in triplicate. A, Relative expression of *ARG1*; B, Relative expression of *iNOS*; C, Relative expression of *NOX2*. Representative data from three separate experiments are shown. *P < 0.05, ***P < 0.001

In this experiment, high doses of oxaliplatin or gemcitabine induced cell death in about half of MDSCs, so we used the less toxic concentrations determined above. The low dose of each agent was the maximum concentration at which the viability curve shown in Figure 2 reached a plateau. Gemcitabine, which is a known agent for the selective depletion of MDSCs, did not significantly reduce the expression of *ARG1*,* iNOS*, or *NOX2* in MDSCs at either a high or low dose (Figure 4A‐C). Interestingly, the low dose of gemcitabine even enhanced *iNOS* expression. In contrast, when MDSCs were treated with the high dose (1 μg/mL) of oxaliplatin, *ARG1* and *NOX2* expression was reduced. Treatment with a low dose (0.03 μg/mL) of oxaliplatin also significantly decreased the mRNA levels of *NOX2* in MDSCs, though the effect was weaker than that of the high dose of oxaliplatin. Although treatment with a high dose of oxaliplatin also led to a mild increase in *iNOS* expression in MDSCs, this was not significant over repeated experiments. These data suggest that the less cytotoxic dose of oxaliplatin may regulate the immunosuppressive function of MDSCs, which was not observed for all cytotoxic drugs.

### 3.5 Changes in MDSC surface markers induced by oxaliplatin

**Figure 5:**
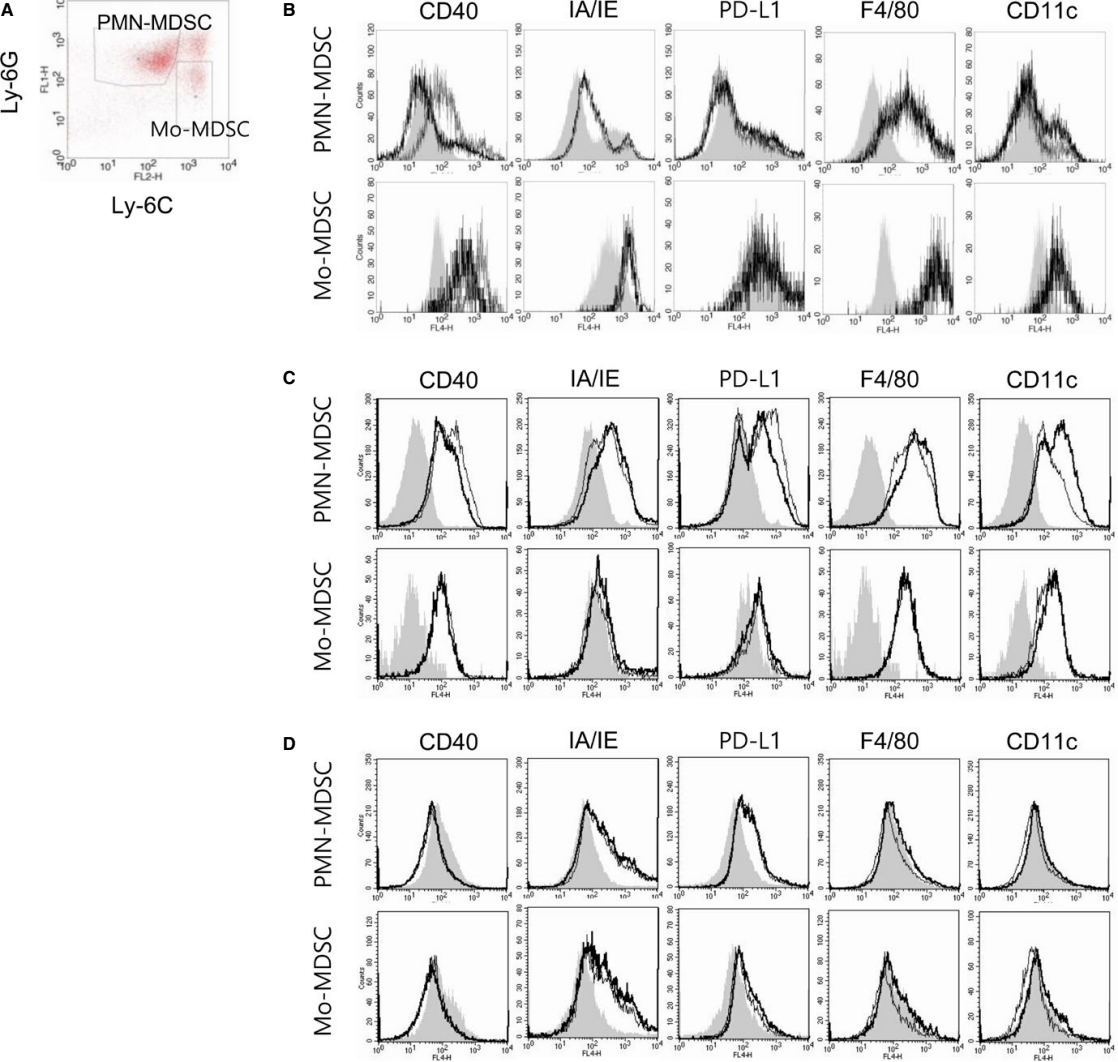
In vitro and in vivo treatment with oxaliplatin resulted in phenotypic changes in myeloid‐derived suppressor cells (MDSCs). For in vitro treatment, CD11b^+^ myeloid cells from the splenocytes of CT26 tumor‐bearing mice were incubated with 1 μg/mL oxaliplatin for 24 h in the presence of 100 ng/mL LPS. For in vivo treatment, CT26 tumor‐bearing mice (n = 3/group) were ip injected with 10 mg/kg of oxaliplatin or PBS. Two days later, mice were sacrificed and cells were stained with fluorescent‐labeled Abs. To classify MDSC subsets, anti‐Ly‐6G Abs conjugated with FITC and anti‐Ly‐6C Abs conjugated with PE were used. Changes in the cosignaling molecules and differentiation markers of MDSCs were determined by flow cytometry. Cells were prepared in triplicate. A, Two subsets of MDSCs. B‐D, Histogram showing the expression levels of surface molecules on each subset. B, Phenotypes of oxaliplatin‐treated MDSCs in vitro; C, Phenotypes of splenic MDSCs from oxaliplatin‐treated tumor‐bearing mice; D, Phenotypes of tumor MDSCs from oxaliplatin‐treated tumor‐bearing mice. Gray fill, isotype control; solid line, vehicle treatment; bold line, oxaliplatin treatment. Representative data from two separate experiments are shown

One of the strategies for reducing accumulated MDSCs is promoting MDSC maturation into macrophages or DCs. To assess MDSC maturation status, MDSC surface molecules were detected by flow cytometry. After 24 hours of incubation with the less cytotoxic dose (1 μg/mL) of oxaliplatin or vehicle, CD11b^+^ MDSCs were stained with fluorescent Abs. Among the CD11b^+^ cells, PMN‐MDSCs were gated as Ly‐6C^int^Ly‐6G^high^ cells, while Mo‐MDSCs were gated as Ly‐6C^high^Ly‐6G^low^ cells (Figure 5A). Interestingly, CD40 expression was reduced by oxaliplatin treatment in both MDSC subsets; however, the expression of IA/IE, which indicates antigen (Ag)‐presenting capacity, and PD‐L1, which is a functional molecule that regulates T‐cell responses,^23^ was not affected by oxaliplatin treatment (Figure 5B). To determine the maturation status of MDSCs, we analyzed levels of a DC‐specific marker, CD11c,^24^ and a macrophage‐specific marker, F4/80,^25^ in each MDSC subset. Following oxaliplatin treatment, levels of CD11c were higher on PMN‐MDSCs but not on Mo‐MDSCs compared with levels following vehicle treatment.

Next, we analyzed the phenotypic changes of MDSCs after in vivo oxaliplatin treatment in tumor‐bearing mice. Consistent with changes induced by in vitro treatment, downregulation of CD40 expression was found in splenic MDSCs (Figure 5C). Interestingly, the levels of IA/IE and CD11c on MDSCs were increased by oxaliplatin treatment. This suggests that treatment with oxaliplatin might influence the immunogenicity of MDSCs. In MDSCs at the tumor site, phenotype changes were less significant than in splenic MDSCs; however, we found the increase in expressions of maturation markers, F4/80 and CD11c on the surface of MDSCs induced by oxaliplatin treatment (Figure 5D). Collectively, oxaliplatin modulated the phenotypes of MDSCs, and these changes may alter the contact‐dependent function of MDSCs.

### 3.6 Oxaliplatin regulates MDSC‐mediated immunosuppression in vitro and in vivo

**Figure 6:**
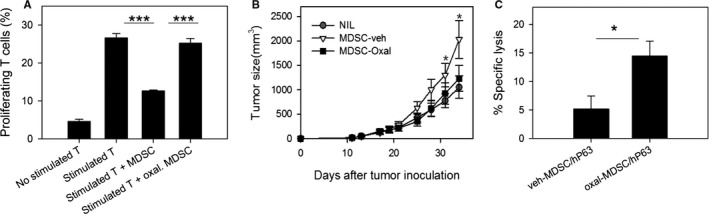
Oxaliplatin treatment weakened the suppressive activity of myeloid‐derived suppressor cells (MDSCs). A, MDSCs isolated from CT26 tumor‐bearing mice were treated with 1 μg/mL oxaliplatin for 4 h in the presence of 100 ng/ml LPS. CFSE‐labeled DO11.10 CD4^+^ T cells were stimulated with 10 μg/mL OVA peptide and cocultured with MDSCs at a 1:1 ratio for 72 h. After incubation, CFSE dilution was assessed for analysis of T‐cell proliferation (n = 3/group). Representative data from two separate experiments are shown. ***P < 0.001, B, Splenic MDSCs from CT26 tumor‐bearing mice were incubated with 1 μg/mL oxaliplatin or sterile water for 2 h in the presence of 100 ng/mL LPS. Then, 1 × 10^7^ MDSCs/mouse were adoptively transferred into BALB/c mice (n = 5/group) that had been sc challenged with 2 × 10^5^ CT26 tumor cells 14 days prior. Tumor size was monitored three times per week. *P < 0.05, MDSC‐veh compared to NIL group. C, MDSCs were incubated in hP63 and oxaliplatin‐containing media for 2 h. As a control, MDSCs were pulsed with hP63 in vehicle‐containing media. After incubation, CTL peptide‐pulsed MDSCs were injected into naïve mice (n = 3/group). Thirteen days later, hP63‐specific target lysis was analyzed by flow cytometry. *P < 0.05

To confirm the immunomodulatory function of oxaliplatin, we compared the activity of oxaliplatin‐treated MDSCs with that of control MDSCs in terms of T‐cell proliferation. CFSE‐labeled DO11.10 T cells stimulated with OVA peptides proliferated during incubation, whereas coculture with MDSCs significantly inhibited T‐cell proliferation (Figure 6A). The MDSC suppression of T‐cell proliferation was neutralized by 4 hours of oxaliplatin treatment.

Next, we analyzed the tumor‐promoting activity of MDSCs in tumor‐bearing mice. It was previously reported that tumor growth rates are accelerated by MDSC adoptive transfer.^26^ Control MDSCs or oxaliplatin‐treated MDSCs were transferred into tumor‐bearing mice, and tumor growth was assessed (Figure 6B). As expected, control MDSCs augmented tumor growth in tumor‐bearing mice. However, oxaliplatin‐treated MDSCs did not lead to increases in tumor size, so that the tumor growth of recipients of oxaliplatin‐treated MDSCs was not different from that of the control group. Therefore, oxaliplatin treatment eliminates the suppressive function of MDSCs, so that oxaliplatin‐treated MDSCs are no longer immunosuppressors or tumor promotors.

We hypothesized that oxaliplatin treatment might induce the conversion of MDSCs from immune suppressors to immune effectors, based on the data which showed the upregulation of CD11c expression on MDSCs (Figure 5). To analyze the immunogenicity of oxaliplatin‐treated MDSCs, we added the CTL epitope peptide of Her‐2/neu tumor Ag (hP63) to MDSCs during oxaliplatin treatment and injected these MDSCs into naïve mice. Interestingly, we found that oxaliplatin‐treated MDSCs could induce slight but significant CTL target lysis in immunized mice (Figure 6C). This suggests that oxaliplatin treatment may render MDSCs less suppressive and more immunogenic.

### 3.7 Oxaliplatin modulates NF‐κB phosphorylation induced by LPS treatment

**Figure 7:**
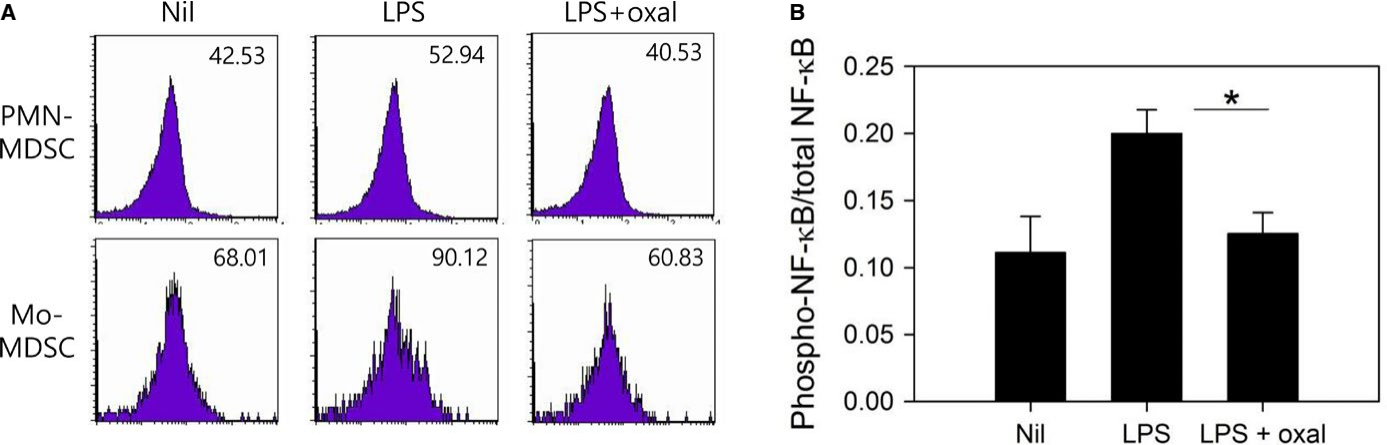
Oxaliplatin modulated intracellular NF‐κB signaling in LPS‐treated myeloid‐derived suppressor cells (MDSCs). A, MDSCs isolated from CT26 tumor‐bearing mice were stimulated with 100 ng/mL LPS in the presence or absence of oxaliplatin for 4 h. Levels of phosphorylated NF‐κB in Ly‐6C^high^Ly‐6G^low^ MDSCs and Ly‐6C^int^Ly‐6G^high^ MDSCs were detected by flow cytometry. Mean fluorescence intensities (MFIs) are shown. Each sample was stained in duplicate. Representative data from two independent experiments are shown. B, MDSCs were stimulated with 100 ng/mL of LPS with or without oxaliplatin for 30 min. NF‐κB p65 total protein and phosphorylated NF‐κB p65 (pS536) were measured by ELISA in cell lysates. *P < 0.05

Recently, it was reported that oxaliplatin‐induced NF‐κB activation in a pancreatic cancer cell line and that this may be linked with cancer drug resistance.^27^ We hypothesized that oxaliplatin may regulate immunosuppressive MDSCs through the positive or negative modulation of signaling pathways, such as the NF‐κB pathway. To analyze the effect of oxaliplatin on the signaling pathways of MDSCs, we detected the phosphorylation of NF‐κB in LPS‐activated MDSCs. Using flow cytometry, we could analyze the level of NF‐κB activation in each subset of MDSCs. LPS activation induced an increase in phosphorylated NF‐κB, and treatment with oxaliplatin resulted in the downregulation of phosphorylated NF‐κB, especially in Mo‐MDSCs (Figure 7A). To confirm modulation of NF‐κB signaling by oxaliplatin treatment, we performed an additional experiment, ELISA. Consistent with flow cytometry, the ratio of phosphorylated NF‐κB/total NF‐κB was increased in LPS‐treated total MDSCs, and oxaliplatin treatment induced the downregulation of NF‐κB activation (Figure 7B). These data suggest that the oxaliplatin‐induced regulation of MDSC‐mediated immunosuppression may be accomplished via the NF‐κB pathway.

## 4 DISCUSSION

Recently, several classes of drugs have been proposed to act as immunomodulators, though their main mechanism of action does not involve the immune system. For example, some antibiotics, in particular those that bind to the ribosome, exhibit interesting effects on immune cells. Macrolides such as azithromycin or clarithromycin modulate CD4^+^ T cells by inhibiting mTOR,^28^ and the inhibitory effect of clarithromycin is restricted to Th2 responses.^29^ Statin is used in dyslipidemia to inhibit 3‐hydroxy‐3‐methylglutaryl (HMG)‐CoA reductase, which is the rate‐limiting enzyme of cholesterol biosynthesis. However, HMG‐CoA reductase is also involved in isoprenoid synthesis, which is required for the small GTPase signaling pathway. Hence, statin has anti‐inflammatory activity against both the adaptive and innate immune responses, and its immunoregulatory function may be of use in cardiovascular disease prevention, together with lowering cholesterol levels.^30^ In addition, anticancer agents may induce the activation of immune cells directly.^2^ Some anticancer drugs deplete proliferating immune cells at cytotoxic doses, while they augment the immunogenicity of immune cells at sublethal doses.^3^, ^4^

Chemotherapeutic agents are administered to cancer patients to eliminate cancer cells but are also known to generate adverse effects, including the depletion of dividing immune cells. Herein, we have examined the effect of anticancer drugs at sublethal doses on the immune systems of tumor bearing mice. The tumor‐induced immunosuppressive environment is known to be one of the barriers to anticancer therapy; therefore, the recovery of immunity in cancer patients is an important issue.

It was reported that treatment with oxaliplatin affected the immunogenicity of DCs.^9^, ^10^ Based on these results, we hypothesized that oxaliplatin might modulate myeloid lineage immunosuppressor MDSCs and play a role in overcoming immunosuppression in the cancer environment. It had already been reported that gemcitabine, doxorubicin, and 5‐fluorouracil (5‐FU) selectively removed MDSCs,^1^ and furthermore, a recent study demonstrated that cisplatin reduced the prevalence and inhibitory function of MDSCs.^31^ However, the immunomodulatory function of oxaliplatin and its molecular mechanism had previously not been reported. In this study, we focused on the immunomodulatory role of oxaliplatin in MDSC‐mediated immunosuppression.

Due to the cytotoxic activity of oxaliplatin, we first confirmed its effect on the depletion of MDSCs in tumor‐bearing mice. Similar to gemcitabine, oxaliplatin selectively depleted MDSCs but not T‐cell populations. We found that Mo‐MDSCs were depleted more efficiently by oxaliplatin treatment than PMN‐MDSCs. In this study, we showed that gemcitabine was more potent in MDSC depletion than oxaliplatin; however, the number of treatments and dosage of the drug also affects the potency of each agent.

In addition, at less cytotoxic doses of the chemotherapeutic agents, oxaliplatin downregulated the expression of the functional mediators of MDSCs, while gemcitabine did not. Levels of *ARG1* and *NOX2* were reduced by oxaliplatin treatment, resulting in the neutralization of the immunosuppression and tumor‐promoting activity of MDSCs. Therefore, we confirmed the immunomodulatory effect of oxaliplatin on MDSC activity.

Moreover, phenotypic changes were observed in oxaliplatin‐treated MDSCs compared with control MDSCs. Oxaliplatin‐treated MDSCs exhibited reduced expression of CD40 and increased expression of CD11c. CD40 is generally known as a marker of activation on immune cells and one of the immune stimulatory receptors. However, it has been reported that surface CD40 on MDSCs mediates an interaction with the CD40 ligand on CD4^+^ T cells and that the CD40‐CD40 ligand interaction leads to differentiation into Treg cells.^32^ Therefore, CD40 may be an immunosuppressive functional molecule on MDSCs. On the other hand, CD40L‐expressing mast cells could render CD40‐expressing PMN‐MDSCs immunosuppressive through CD40L/CD40 interaction in prostate cancer.^33^ This suggests that CD40 on MDSCs may be important for MDSCs becoming immunosuppressive cells. Besides, it was reported that high level of CD40 expression on MDSCs correlated with upregulation of CXCR5 and promoted the recruitment of MDSCs to the cancer site.^34^ A recent study demonstrated that decreased CD40 expression on MDSCs correlated significantly with MDSC accumulation in gastric tumor‐bearing mice and CD40 activation using anti‐CD40 agonistic Abs induced the apoptosis of MDSCs.^35^ Therefore, further studies are required to elucidate the effect of downregulation of CD40 on MDSCs after oxaliplatin treatment. CD11c is a DC differentiation marker found on myeloid lineage cells. In the cancer environment, MDSCs accumulate as immature cells and exhibit a suppressive function. However, enforced maturation of MDSCs results in a reduction in immunosuppressive activity and the conversion of suppressive cells into immunogenic myeloid cells.^36^ Under the proper conditions, MDSCs can differentiate into DCs or macrophages.^37^ Although CD11c expression alone does not demonstrate the maturation of MDSCs into DCs, it does indicate a phenotypic change in MDSCs, and the upregulation of CD11c suggests the possibility that the further maturation of MDSCs was induced by oxaliplatin treatment. If oxaliplatin does contribute to the maturation of MDSCs, differentiated cells could play a role as immune effectors and mediate anticancer immune responses in cancer patients.

The basic molecular mechanism of oxaliplatin as a cytotoxic chemotherapeutic agent involves binding to double‐stranded DNA and inhibiting DNA replication and transcription. However, the immunomodulatory activity of oxaliplatin at a less toxic dose may be derived from a distinct mechanism. One of the mechanisms of chemoresistance in cancers is the activation of signaling molecules, including MYC and AKT1,^38^ and the ability of some chemotherapeutic agents to restore the sensitivity of drug‐resistant cancer cells may be related to their modulatory effect on certain signaling pathways, such as STAT3.^39^ Therefore, we hypothesized that the mechanism by which oxaliplatin regulates MDSC‐mediated immunosuppression may involve the modulation of key signaling pathways in MDSCs. STATs are recognized as functional and developmental signaling regulators of MDSCs,^40^ and it has also been reported that STAT3 phosphorylation is inhibited by oxaliplatin treatment in a cancer cell line.^39^ However, we did not detect significant inhibition of STAT3 phosphorylation in oxaliplatin‐treated MDSCs (data not shown). Another candidate for the molecular targeting of oxaliplatin was NF‐κB. We had already confirmed that treatment with LPS stimulated the NF‐κB pathway in MDSCs,^14^ and it was reported that oxaliplatin treatment affected NF‐κB signaling in a cancer cell line.^27^ In the present study, we found that oxaliplatin treatment blocked NF‐κB activation in LPS‐stimulated MDSCs. Thus, the data suggest that oxaliplatin regulates the suppressive function of MDSCs via the downregulation of NF‐κB signaling activation.

Generally, cytotoxic drugs, such as gemcitabine and 5‐FU, have been found to selectively eliminate MDSCs, and combined with other immunotherapy, they improve the therapeutic effect.^16^, ^41^, ^42^ It has been reported that docetaxel induced selective depletion of the mannose receptor (MR)^+^ MDSCs, resulting in M1‐like MDSCs accumulation in tumor‐bearing mice.^44^ In addition to reduction of MDSCs, some agents modulate the suppressive function of MDSCs. All‐trans‐retinoic acid (ATRA) induced maturation of MDSCs and downregulated the functional mediator, reactive oxygen species (ROS).^45^ It was reported that the PDE‐5 inhibitor, sildenafil, downregulated the suppressive function of MDSCs.^46^ Collectively, the modulatory effect of drugs on MDSCs is based on depletion, maturation, or functional inhibition of MDSCs. However, recent reports have demonstrated the stimulatory effects of chemotherapeutic drugs on MDSCs. Cytotoxic drugs, gemcitabine and 5‐FU, modulate MDSCs by activating the Nlrp3 inflammasome and limit the antitumor efficacy of these chemotherapeutic agents.^47^ It was also reported that low dose cyclophosphamide treatment induced MDSC expansion and activation.^48^ Therefore, it is important to identify the effects and mechanisms of chemotherapeutic drugs on MDSCs, because appropriate drug treatment results in improvement of therapeutic effects in cancer patients.

Myeloid‐derived suppressor cells represent one of the major types of regulatory cells, and they contribute to establishing a suppressive environment in cancer‐bearing hosts. In the present study, we found that oxaliplatin regulated MDSCs via depletion at high doses or functional modulation at less cytotoxic doses. Chemotherapeutic agents generally target MDSCs via cytotoxic activity, but they also may have the capacity to regulate MDSCs by subverting intracellular signaling transduction. Our study suggests new strategies for MDSC regulation using oxaliplatin. Following further investigation, oxaliplatin, which has been widely used as a cytotoxic drug, may be used as an immunomodulator in cancer patients.

## CONFLICT OF INTEREST

The authors declare no conflict of interests.
