# Supplementary figures and images for: Oxaliplatin regulates myeloid‐derived suppressor cell‐mediated immunosuppression via downregulation of nuclear factor‐κB signaling
Source: Cancer Med. 2018 Dec 27;8(1):276–88. doi: 10.1002/cam4.1878 (PMC6346236; doi:10.1002/cam4.1878)

Supplementary Figure 1

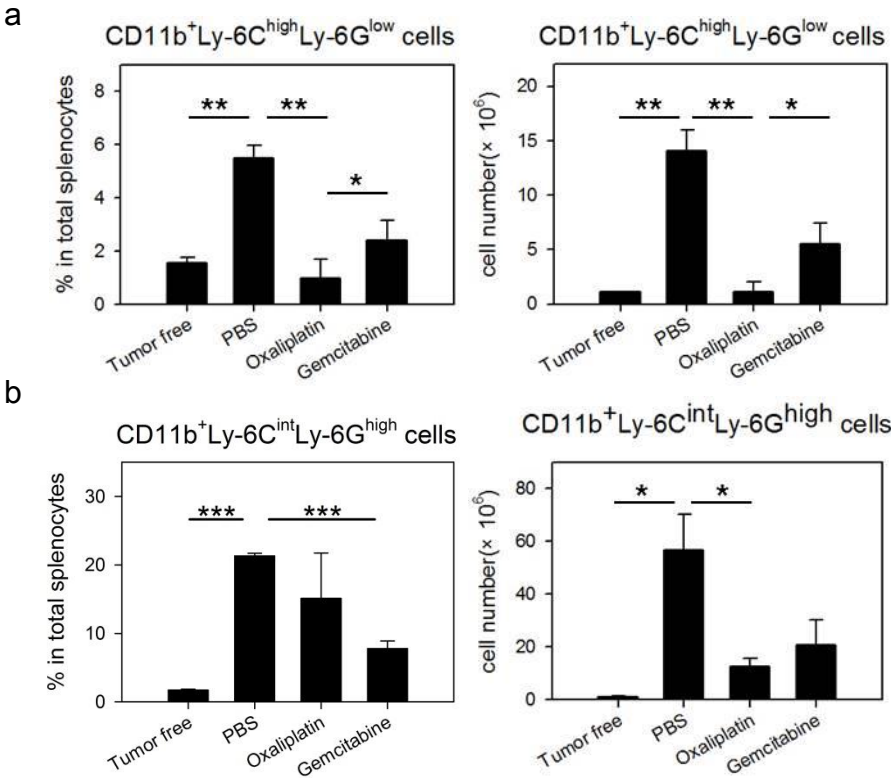

Supplementary Figure 2

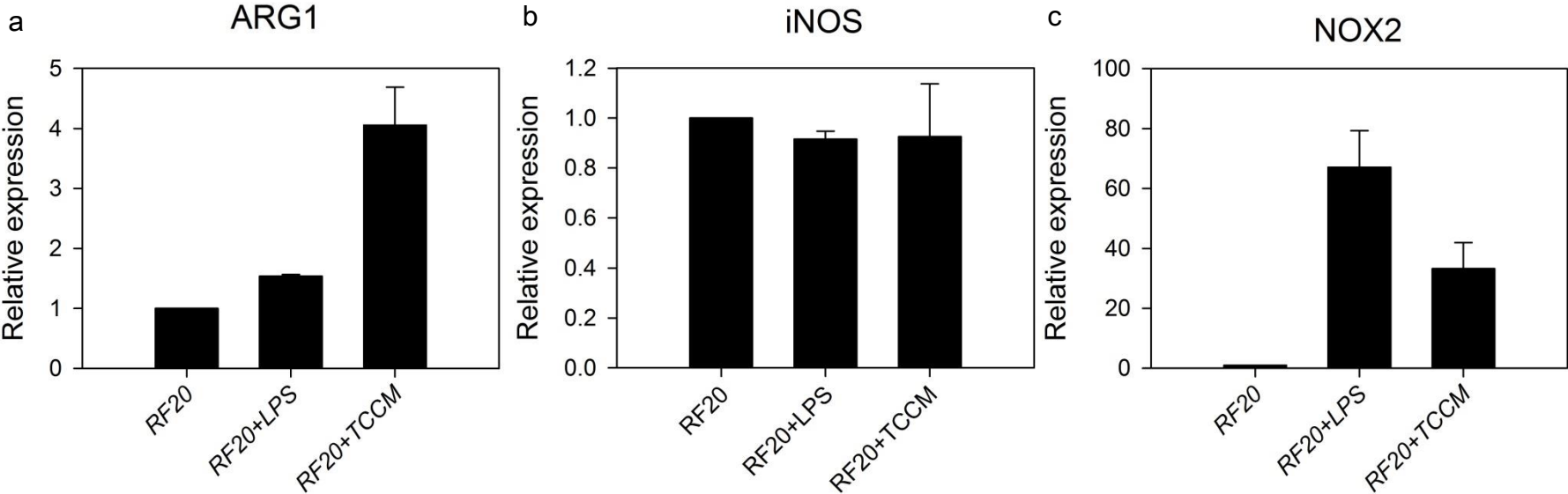

Supplement: Supplementary file 1 [file CAM4-8-276-s001.pdf]
