# Supplementary material for: Oxaliplatin regulates myeloid‐derived suppressor cell‐mediated immunosuppression via downregulation of nuclear factor‐κB signaling
Source: Cancer Med. 2018 Dec 27;8(1):276–88. doi: 10.1002/cam4.1878 (PMC6346236; doi:10.1002/cam4.1878)
Supplement: Supplementary file 2 [file CAM4-8-276-s002.docx]

**Supplementary figure 1. *In vivo* treatment with oxaliplatin selectively removed MDSCs in tumor-bearing mice.**

BALB/c mice were s.c. inoculated with 1×10^5^ CT26 cells/mouse. When the average tumor size reached approximately 1000~1200 mm^3^, oxaliplatin or gemcitabine was i.p. injected into tumor-bearing mice. In oxaliplatin treatment group (n=3), mice were injected 10 mg/kg oxaliplatin twice at the 2 days interval, while the other tumor bearing mice (n=-3) were treated with 120 mg/kg gemcitabine once at the day when the second treatment with oxaliplatin was performed. As control, PBS was i.p. injected into the remained tumor bearing mice (n=3). Two days after the last drug treatment, T cells and MDSC subsets among total splenocytes were analyzed by flow cytometry. (A) Percentages (left) and absolute numbers (right) of CD11b^+^Ly-6C^high^Ly-6G^low^ cells in splenocytes. (B) Percentages (left) and absolute numbers (right) of CD11b^+^Ly-6C^int^Ly-6G^high^ cells in splenocytes. **p* < 0.05, ***p* < 0.01, ****p* < 0.001.

**Supplementary figure 2. Either LPS or tumor conditioned culture media (TCCM) increased suppressive activities of MDSCs.**

CD11b^+^ cells were purified from the splenocytes of CT26 tumor-bearing mice, and treated with 100μg/ml LPS or TCCM. To generate TCCM, CT26 cells were cultured in RPMI 1640 medium with a 3% FBS for 48 h. TCCM were obtained by adding 20 ng/ml of GM-CSF and 30% culture supernatant in RPMI1640 medium with a 20% FBS. After 24 h of treatment, total RNA was extracted from MDSCs and used as a template for cDNA synthesis. Quantitative PCR was performed to analyze the mRNA levels of *ARG1*, *iNOS*, and *NOX2*. (A) Relative expression of *ARG1*. (B) Relative expression of *iNOS*. (C) Relative expression of *NOX2*. Representative data from two separate experiments are shown.
